# Microbial diversity and nitrogen cycling across oxygen gradients in the eastern tropical Pacific during two La Niña years

**DOI:** 10.1128/msystems.00893-26

**Published:** 2026-09-21

**Authors:** Peihang Xu, Damian L. Arévalo-Martínez, Mathias Middelboe, Marei Pohlmann, Hermann W. Bange, Carolin R. Löscher

**Affiliations:** 1 Department of Biology, University of Southern Denmark 6174, Odense, Denmark; 2 Leibniz Institute for Baltic Sea Research Warnemünde 28389, Rostock, Germany; 3 Chemical Oceanography, GEOMAR Helmholtz Center for Ocean Research Kiel, Kiel, Germany; 4 Department of Biology, University of Copenhagen 4321, Copenhagen, Denmark; 5 Danish Center for Hadal Research, University of Southern Denmark 6174, Odense, Denmark; 6 Danish Institute for Advanced Science (DIAS), University of Southern Denmark 6174, Odense, Denmark; University of Illinois Chicago, Chicago, Illinois, USA

**Keywords:** El Niño, La Niña, oxygen minimum zone, nitrification, denitrification, anammox, metagenome, tara oceans, eastern tropical Pacific

## Abstract

The rapid expansion of ocean oxygen minimum zones (OMZs) may significantly affect the microbial processes that regulate marine nitrogen cycling. The eastern tropical Pacific (ETP) bears one of the largest perennial OMZs, which is strongly influenced by the recurring El Niño Southern Oscillation (ENSO). However, how microbial diversity and nitrogen cycling respond to oxygen variability under comparable ENSO conditions remains unclear. Here, we applied metagenomics to analyze changes in microbial communities across a dissolved oxygen (DO) gradient from oxic to suboxic conditions in the ETP during the 2022 La Niña event and compared our findings to the *Tara Oceans* data set from the same region during the 2011 La Niña. In 2022, we observed a decline in microbial alpha diversity and abundance, coupled with an increase in nitrogen metabolism genes as DO decreases. The relative abundance of many dominant microbes shifted at a DO threshold of 80 μmol kg^−1^, while dominant nitrogen cycle genes varied under different DO thresholds (120, 20, 5 μmol kg^−1^). Despite the 11-year interval between sampling efforts, microbial alpha diversity was similar between 2011 and 2022. We observed a significant reduction of SAR11 and an increase of Poseidoniia in 2022 compared with 2011. While certain nitrogen cycling genes differed in relative abundance in a depth-dependent manner, overall functional group composition was largely consistent, with spatial variation exceeding interannual differences. Overall, our study presents a comprehensive examination of possible shifts of microbial diversity, community, and their potential for nitrogen cycling under deoxygenation during two La Niña years.

**IMPORTANCE:**

The eastern tropical Pacific oxygen minimum zones (OMZs), largely impacted by the natural climate cycle—El Niño-Southern Oscillation, are predicted to continually expand while their core may shrink. While deoxygenation is known to profoundly influence microbial ecosystems, how microbial diversity, composition, and nitrogen cycling would shift across oxic/hypoxic/suboxic gradients, and its association with ENSO dynamics remains poorly understood. We surveyed microbial communities under two La Niña years with an 11-year gap and found that vertical variability is more critical than temporal variations for both microbial diversity and microbe-mediated nitrogen pathways, as only slight differences in microbial diversity and nitrogen cycles were detected between the two studied years despite ENSO dynamics, which might have disrupted the system among the 11 years. Moreover, we identified oxygen thresholds causing dominant microbes and potential nitrogen pathways to shift, thus helping to improve prediction on how various microbes and nitrogen pathways might respond to further deoxygenation.

## INTRODUCTION

Oxygen minimum zones (OMZs), characterized as oceanic subsurface waters with low oxygen concentrations, have expanded rapidly over the global ocean both vertically and horizontally in the past five decades (1–3). This expansion of OMZs is associated with increasing ocean temperatures, which reduce oxygen solubility in seawater, increased sinking of organic matter, which fosters biological oxygen demand at depth, and reduced ventilation caused by increased stratification (3, 4). When the concentration of dissolved oxygen (DO) drops below ~60 to 120 μmol kg^−1^, most aerobically respiring macroorganisms experience stress or even die (5, 6). Many microbes can, however, thrive under low-oxygen conditions and are active even under suboxic (DO ≤ 5 µmol kg^−1^) and anoxic (DO ≤ 10 nmol kg^−1^) conditions, playing a unique role in the cycling of nutrients and other elements, as well as the turnover of climate-relevant trace gases (e.g., N_2_O) in OMZs (6, 7).

Oxygen gradients in OMZs create intricate habitats that support a dynamic microbially mediated nitrogen cycle (4, 8). Permanent ocean OMZs, including the eastern tropical South Pacific Ocean (ETSP), the eastern tropical North Pacific Ocean (ETNP), the Bay of Bengal (BoB), and the Arabian Sea (AS), are ideal settings to study those complex microbial processes and their impacts on a global scale (7, 9–12). Nitrification is initiated by ammonia monooxygenase (encoded by *amo*ABC) in ammonia-oxidizing archaea (AOA) and bacteria, followed by nitrite oxidation via nitrite oxidoreductase (*nxr*AB). While these organisms are typically associated with oxygenated waters (4), AOA are also found in hypoxic and anoxic OMZs (13, 14), potentially because some AOA species can produce oxygen under anoxic conditions (15). Nitrite oxidation can also persist under low-oxygen concentrations, even influencing nitrite accumulation within OMZ (16). Under suboxic or anoxic conditions, nitrate reduction to nitrite (*nar*GH) can initiate denitrification, followed sequentially by nitrite reduction (*nir*S or *nir*K), nitric oxide reduction (*nor*B), and N_2_O reduction (*nos*Z) to N_2_ (17). Other anaerobic pathways include anaerobic ammonium oxidation to N_2_ (anammox), mediated by hydrazine dehydrogenase (*hdh*), and dissimilatory nitrate reduction to ammonium (DNRA), indicated by *nrf*AH (8). Although denitrification and anammox have been traditionally associated with suboxic or anoxic waters (3, 18, 19), recent studies challenge a strict separation between oxygenated and anaerobic nitrogen cycling, suggesting that denitrification might occur in anaerobic niches of particles in hypoxic waters with DO of 5–60 μmol kg^−1^ (20). The terminal step of denitrification, the reduction of N_2_O to N_2_, which has classically been regarded as highly sensitive to oxygen, has also been found to be potentially active in oxic waters, as indicated by the presence and expression of *nos*Z in the ETSP and AS OMZs (21, 22). Together, these observations suggest that nitrogen cycling processes are not strictly separated between oxic and anoxic processes but instead occur and respond differently across a broad oxygen gradient. Low-oxygen transition zones may therefore represent distinct microbial niches in which multiple, and potentially competing, nitrogen transformations coexist before the onset of strongly anoxic conditions. As low-oxygen waters are projected to expand with ongoing ocean deoxygenation (23, 24), understanding how microbial nitrogen-cycling potential responds across these non-anoxic oxygen gradients is critical for predicting future changes in nitrogen loss and N_2_O cycling in OMZs.

The eastern tropical Pacific Ocean (ETP) harbors the two largest OMZs (ETSP and ETNP) and encompasses one of the four major eastern boundary upwelling systems (the Peru upwelling system) in the global ocean (24, 25). Microbial processes and nitrogen cycling within the ETSP and ETNP OMZs have been extensively investigated, revealing these systems to be major hotspots for microbial activity in low-oxygen waters, microbially mediated nitrogen loss, and intense N_2_O and CO_2_ emission to the atmosphere (22, 26–29). In contrast, the broader ETP region, which represents a low-oxygen transition between the two major ETSP and ETNP OMZs, remains comparatively understudied and lacks comprehensive data sets.

How deoxygenation (understood here as a decrease in dissolved oxygen concentrations) impacts the microbial community, and their functions involved in nitrogen cycling in the Pacific OMZs, have been highlighted in several studies, with a particular focus on anaerobic niches (30, 31). However, while the ETP OMZs have been predicted to expand (2), more recent studies show that their core waters—where DO is lowest (5–20 µmol kg^−1^)—may shrink (24). Hence, it is important to understand the shifts of microbial diversity, composition, and their potential contributions to key biogeochemical cycles across oxic/hypoxic/suboxic niches in the Pacific OMZs to allow predictions of the effects of the ongoing changes of OMZ distribution and oxygen characteristics on biogeochemical cycling in these important marine environments. To address this, we applied a gene-centric metagenomic approach (7, 32) to systematically examine spatial variations in microbial diversity and nitrogen cycling potential across a gradient from well-oxygenated to the suboxic waters of the ETP. Functional genes provide markers of genetic potential for specific nitrogen transformations: presence, absence, and relative abundance of genes such as *amo*A, *nir*S, *nos*Z, and *hdh* allow us to assess which pathways are encoded by the community and how these potential changes along the oxygen gradient. Despite the limitation that metagenomics reflects genetic potential rather than activity, it provides a robust framework for investigating microbial diversity and functional capacity.

The ETP is also affected by a naturally occurring climate cycle—the El Niño-Southern Oscillation (ENSO), which modulates upwelling intensity and the OMZ upper boundary (33–35). During La Niña (El Niño) conditions, the Pacific OMZs experience upper boundary shoaling (deepening), lower (higher) sea surface temperature, intensified (suppressed) upwelling (36, 37). Such physical perturbations between La Niña and El Niño not only alter denitrification rates, primary production, and N_2_O accumulations in the water column (34–36) but also restructure microbial assemblages and nitrogen transformation pathways that underpin these biogeochemical responses (38, 39). However, although previous studies have highlighted ENSO-driven contrasts in microbial community composition, particularly during strong El Niño events, the stability and reproducibility of microbial responses across different La Niña events over decadal timescales remain poorly constrained. Microbial assemblages is an informative indicator of ecosystem responses to environmental variability, including oxygen and nutrient (40, 41). Here, we compared the metagenome data sets from this study (2022) with earlier data sets from the *Tara Oceans* expedition (2011) (42) from the same ETP region, enabling an assessment of the potential temporal variability in microbial composition and nitrogen cycling potential under comparable ENSO phases—association with two La Niña events separated by an 11-year interval. During this period, one strong El Niño occurred in 2015–2016, along with several moderate to weak El Niño and La Niña events in the ETP (https://psl.noaa.gov/enso/mei/). We hypothesized that, given broadly comparable environmental conditions, prokaryotic community assembly and the distribution of nitrogen cycling genes remain relatively consistent between the two La Niña years. Such stability would suggest that the microbial community is relatively resilient to recurring La Niña conditions even as upwelling intensity fluctuates, with the genetic capacity for the major nitrogen transformations—including nitrification, denitrification, anammox, and DNRA—being largely conserved, together with the potential for fixed-nitrogen removal and N_2_O production. Conversely, substantial shifts in community composition or functional genes would indicate sensitivity to interannual variability, with potential consequences for the balance among oxidative and reductive nitrogen pathways and the partitioning of nitrogen between N_2_ and N_2_O.

## MATERIALS AND METHODS

### Sampling and measurement of environmental parameters

**Fig 1:**
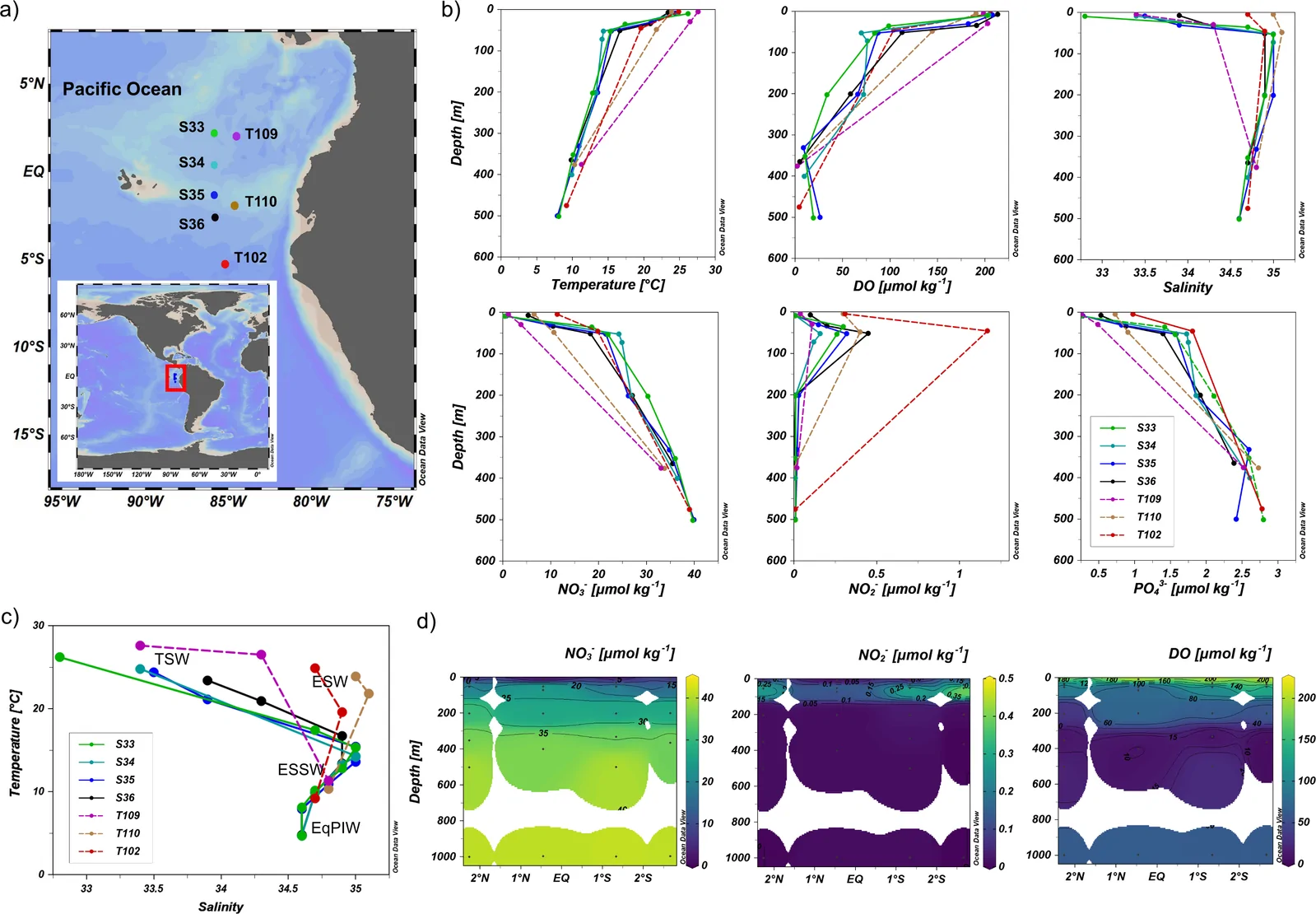
**(a**) sampling stations in the same sampling regions of ETP between the two cruise periods, SONNE 287 cruise (S33, S34, S35, S36) and *Tara Oceans* (T102, T109, T110); (**b**) comparison of depth profiles of physicochemical variables (temperature, DO, salinity, NO_3_^−^, NO_2_^−^, PO_4_^2−^) between the cruise periods; (**c**) T-S diagram for both cruise periods in the ETP. Four water masses were shown: tropical surface water (TSW); equatorial surface water (ESW), equatorial subsurface water (ESSW), and equatorial Pacific intermediate water (EqPIW); (**d**) spatial profiles of physicochemical variables (NO_3_^−^, NO_2_^−^, DO) crossing the sampling regions during SONNE 287 cruise period.

Samples were collected during a cruise on the German research vessel RV SONNE (SO287) to the ETP on 7–8 January 2022. Water samples were collected at four stations in the ETP (stations S33–S36) with five to six depths at each station along 85.83°W from 2.26°N to 2.67°S, close to three sampling sites of the *Tara Oceans* expedition (T109, T110, and T102) (42) along 84.51-85.2°W from 2.08°N to 5.27°S sampled from 21 April to 22 May 2011 (Fig. 1a; Table S1). The samples were obtained from a 10-L Niskin bottle rosette water sampler equipped with a conductivity-temperature-depth (CTD) sensor and an oxygen sensor (Sea-Bird Electronics, Bellevue, WA, USA). The oxygen sensor was calibrated with manual Winkler titration (43). Analyses of nutrients, including nitrite (NO_2_^−^), nitrate (NO_3_^−^), phosphate (PO_4_^3−^), and silicate (SiO_2_), were conducted on board immediately after sampling with a QuAAtro auto-analyzer (SEAL Analytical GmbH, Germany) (44). Water samples for N_2_O measurements were collected and analyzed as described in Kock et al. (43). For DNA samples, 1–2-L seawater was obtained and filtered onto a 0.22-µm MCE membrane filters (Merck Millipore Ltd., Ireland). Filters were flash-frozen in liquid nitrogen and stored at −80˚C until further analysis. Unlike SO287 DNA samples, *Tara Oceans* DNA samples were obtained by pre-filtering seawater to remove larger organisms and collecting prokaryotes onto 0.22−3-µm size fractions, representing microbial communities across different ocean depths and thus largely comparable to our >0.22-µm fraction (42). Then, 1 mL seawater of each sample was fixed with paraformaldehyde (with a final concentration of 0.2%) for the measurement of abundance of heterotrophic prokaryotes and subsequently frozen.

### Abundance of heterotrophic bacteria

Heterotrophic bacteria were counted using a FACSCanto II flow cytometer (Becton Dickinson, CA, USA) following available protocols (45). Samples were diluted 10x in TE buffer, stained with SYBR Green I for 5 min, and run for 30 s at medium flow rate with a threshold set at 500 on green fluorescence. Controls with only SYBR Green I dye, and TE buffer were run as a blank background. Known concentrations of BD Trucount beads (Becton Dickinson, CA, USA) were run every 8–10 samples with the same setting for each population to ensure comparable and normalized parameters.

### DNA extraction, library preparation, sequencing

Microbial DNA was extracted from filters using a Qiagen DNA/RNA AllPrep Kit (Qiagen, Hildesheim, DE) according to the manufacturer’s protocol. The concentration of DNA was quantified spectrofluorometrically on a Qubit 4 Fluorometer (Thermo Fisher Scientific), and the quality of DNA was checked using agarose gel electrophoresis.

The metagenome library preparation and sequencing were conducted as a commercially available service by BGI (China). Sequencing libraries were generated using VAHTS Universal DNA Library Prep Kit for MGI (Vazyme) following the manufacturer’s protocol. Sequencing was performed with 2 × 150 bp on a DNBSEQ-G400 platform (MGI). Low-quality reads, adaptor sequences, and contaminants were removed from the paired-end sequence reads using SOAPnuke1.5.5 (46). Clean reads that passed quality check accounted for 96%–98% of raw reads. Twenty-two metagenomic data sets were generated in total, with an average of 11.8 Gbp (39.3 million clean reads) per sample (Table S2). Nine metagenome data sets with clean reads from the *Tara Oceans* projects were downloaded from the ENA database (https://www.ebi.ac.uk/ena/browser/view) (Table S2). For *Tara Oceans* samples, DNA was extracted using the Acinas laboratory phenol–chloroform protocol, and libraries were prepared following the *Tara Oceans* MetaG protocol (~300 bp inserts) and sequenced on the Illumina HiSeq 2000 (47). To reduce analytical bias, Tara raw clean reads were reprocessed using the same bioinformatic workflow as our SO287 data sets to ensure consistency.

### Taxonomic profiling from metagenome data sets

To assign the taxonomy, Kraken2 (v2.1.3) (48) was used with the GTDB database (release 207, size in 308 G) from a pre-built database by Struo2 (49). The taxonomic reports generated by Kraken2 were uploaded to Pavian (50), an interactive R-based visualization tool for metagenomic data exploration. Within the Pavian Comparison module (enabling cross-sample taxonomic comparison), we selected species-level read counts matrix across all samples to export. Each unique species-level taxon was subsequently treated as an operational taxonomic unit (OTU) for further analysis. Subsequently, two OTU tables were exported; one for the metagenome data sets from this study and one for *Tara Oceans* metagenome data sets. Each table contained read counts of taxonomic assigned microbes that were used for downstream analysis. All reads were rarefied to the lowest reads in each of the two data sets using ampvis2 v2.6.7 (51), which was also performed for heatmaps plots, the analysis of alpha diversity (Chao1, Shannon, and Simpson index), and beta diversity (principal coordinates analysis, PCoA).

### Metagenomic *de novo* assembly, gene prediction, and annotation

Clean sequence reads of each sample data set were assembled using Megahit v1.2.9 (52) with a minimum contig length of 500 bp. Open reading frames (ORFs) of the assembled contigs from each sample data set were predicted using Prodigal v2.6.3 (53) with the “-p meta” options. All ORFs from all the 22 data sets were generated into a catalog of non-redundant genes (cNRG) with a minimum length of 100 bp after clustering using cd-hit-est (>95% sequence identity) (54). The clean reads from each sample data set were mapped against the cNRG using salmon v1.6.0 (55) by applying the “--meta” option and subsequently to generate the genes per million reads (GPM) abundance table. The GPM table contained the normalized non-redundant gene abundance across the 22 metagenomic data sets. Another cNRG and GPM table were also generated for the nine *Tara Oceans* metagenomic data sets, following the above processes. To evaluate the taxonomic and functional profiles of genes, KofamKOALA (56) was performed to annotate the cNRG to assign KEGG Orthology (KO) numbers with the threshold of e-value at 1 × e^−5^, and DIAMOND protein aligner (v2.1.10) (57) was conducted to perform blastp searches of cNRG against NCBI nr (non-redundant) database to identify taxonomy and their titles for each gene. To assign the best hit for specific taxa, threshold values were set at bit score (>50), amino acid similarity (>60), and alignment length (>50) (58). The relative abundance, identified as GPM, of each nitrogen metabolism was calculated based on genes identified to their corresponding titles and KO numbers (Table S3).

### Statistical analysis of environmental variables

Samples were grouped into six DO regimes, ranging at oxic (>120 µmol kg^−1^), low-oxygenated (<120, 80–120, 60–80 μmol kg^−1^), hypoxic (20–60, 5–20 μmol kg^−1^), and suboxic (≤5 µmol kg^−1^) conditions (Table S1), which represent important DO thresholds for biological processes and OMZ expansion (7, 24). For a better comparison of *Tara Oceans* data sets and ours, samples were also categorized into seven depths, including depths at surface (<10 m), 30 m, 50 m, 70 m, 200 m, OMZ core (330–480 m), 500 m (Table S1). The vertical profiles of environmental factors, abundance of heterotrophic prokaryotes (bacteria and archaea), and the sampling map were visualized by Ocean Data View (v 5.6.3). The variance analysis of alpha diversity and nitrogen metabolism between *Tara Oceans* and our samples were analyzed using unpaired *t*-test. Pearson correlation was used for analyzing the association between relative abundance of each functional pathway and variable environmental factors. Linear regression analysis was used for analyzing the correlation between alpha diversity, or the relative abundance of each top family microbial groups, or the GPM of selected genes and DO or N_2_O. The analysis mentioned above was visualized by GraphPad Prism (v9.5.1, United States). Bio-Env analysis (Primer 7, Plymouth, UK) was performed to elucidate the extent of environmental variables contributing to the pattern of microbial composition of the whole community or those involved in nitrogen metabolisms from SO287 expedition using distance-based linear modeling (DistLM) with Marginal tests. A PCoA analysis was conducted to assess the similarity of nitrogen functional groups between samples collected from both SO287 and *Tara Oceans* expeditions. Redundancy analysis (RDA) was additionally performed to identify environmental drivers of nitrogen cycling gene composition when involving both SO287 and *Tara Oceans* data sets.

## RESULTS AND DISCUSSION

### Environmental characteristics

During the sampling period of SO287, the depth profiles of various environmental parameters at stations along ~86°W (2.26°N–2.67°S) were quite similar (Fig. 1d; Fig. S1; Table S1). For example, temperature was high (23.4°C–26.2°C) at the surface and decreased with depth, reaching ~4.7°C at 1,000 m. Salinity was lower in surface waters (32.8–33.9) compared with depths at >50 m (34.6–35). Turbidity decreased with depth (0.05–0.107 NTU at <10–30 m depth to 0–0.027 NTU at 1,000 m). Three water masses were identified according to their characteristic temperature and salinity (35, 59). The tropical surface water (TSW) featured warm temperature and low salinity (T > 23.4°C, S < 34). The equatorial subsurface water (ESSW, T = 9.9–13.6°C, S = 34.7–35) was found at depths of 200–400 m, which during upwelling can impact the water column properties at depths up to 50–70 m (T = 14.2°C–16.7°C, S ≈ 35) (Fig. 1c). The equatorial Pacific intermediate water (EqPIW, T = 4.7°C–8.1°C, S = 34.6) was found at depths of 500–1,000 m.

Nitrate (NO_3_^−^), phosphate (PO_4_^3−^), and silicate (SiO_2_) concentrations increased with depth, ranging from <1 µmol kg^−1^ in the surface to 41.5, 2.93, and 88 μmol kg^−1^ at 1,000 m, respectively (Fig. S1). DO concentrations reached minimum concentrations of 5.2–10.6μmol kg^−1^ within the OMZ core at ~330–400 m (Fig. 1d). The thermocline and oxycline deepened from the north of the Equator (S33, S34) to the south (S35, S36) (Fig. S1). Consistently, higher nutrient concentrations were observed at 30/50m in the northern stations compared with the southern stations, indicating stronger upwelling north of the Equator (Fig. S1, Table S1). A nitrite (NO_2_^−^) maximum (0.1–0.45 μmol kg^−1^) was present at 30–70 m at all the stations. Otherwise, nitrite concentrations ranged between 0 and 0.03 μmol kg^−1^ (Fig. S1 and Table S1). Our stations lie between the ETSP and ETNP OMZs (Fig. 1a); oxygen and nitrite patterns show a low-oxygen transition zone (>5 µmol kg^−1^), lacking the micromolar secondary nitrite maximum (SNM) characteristic of functional anoxic layers in OMZ. Our sampling was conducted in January 2022 during weak La Niña conditions, when the multivariate ENSO index ranged from −0.96 to −1.06 (Fig. S2). The observed low-oxygen transition zone and absence of SNM were consistent with typical conditions of the ETP OMZ along 86°W at the Equator across different ENSO phases (35, 43, 60). However, the upper boundary of the OMZ was shallower during La Niña compared with other phases (Fig. S3) (60). The OMZ core (between 300 and 450 m) is thicker during La Niña periods than during El Niño (60).

Nitrous oxide (N_2_O) reached its maximum concentration at S34 at 70m (62.2 nmol kg^−1^), and at S36 at 200 m (77.9 nmol kg^−1^) (Fig. S1). At the other two stations, N_2_O increased with depth from the surface (8–9.5 nmol kg^−1^) to the OMZ core (56.2–61.9 nmol kg^−1^). The concentrations and vertical distribution of N_2_O within the OMZ core were consistent with previous observations from the same ETP region during La Niña and neutral conditions (35). In contrast, previous studies showed higher N_2_O concentrations (>80 nmol kg^−1^) and a deeper N_2_O maximum in association with El Niño conditions, likely reflecting the deepening of thermocline and OMZ (35, 43).

### Comparing sampling and environmental features between two La Niña years

The general physicochemical profiles of our sampling stations (S33–S36) were similar to the *Tara Oceans* sampling sites (T102, T109, T110) (Fig. 1a), although the *Tara Oceans* sampling was restricted to the upper 10m (surface layer), 30 m, 50 m, and the OMZ core. Both sampling campaigns were conducted during La Niña conditions, but with different phases and persistence: *Tara Oceans* (April–May 2011) occurred during the decaying phase of a strong La Niña (MEI.v2: −1.13 to −1.35), while our sampling (January 2022) occurred during a persistent 3-year La Niña (MEI.v2: −0.96 to −1.06) (Fig. S2) (61). In contrast to the uniform water mass distribution across the four SO287 stations, the *Tara Oceans* stations exhibited spatial heterogeneity. Station T109 shared the same TSW characteristics (T = 25°C, S < 34), whereas stations T102 and T110 were influenced by Equatorial surface water (ESW, T < 25°C, S >34) (Fig. 1) (59).

Subsurface waters (30–50 m) during our campaign constrained higher nutrient concentrations (NO_3_^−^), lower temperature, and DO than those observed during *Tara Oceans*, suggesting stronger upwelling in January 2022. This interpretation is supported by model and satellite data showing shallower oxycline, thermocline, and halocline depths, higher surface *Chla* concentrations but lower sea surface temperature in January 2022 compared with May 2011 (Fig. S3 and S4). Both data sets further indicate enhanced upwelling during La Niña years (2011 and 2022) compared with neutral and El Niño years (2015/2016 and 2019) (Fig. S3 and S4). Increased productivity induced by enhanced upwelling during La Niña has been linked to greater organic matter export and increased microbial respiration in the subsurface waters of the ETP, which can contribute to oxygen consumption and influence OMZ biogeochemistry (62). Although the vertical oxygen gradient persists across different ENSO states, their magnitude, depth, and spatial extent can vary between El Niño, La Niña, and neutral conditions (Fig. S3). The OMZ core was located at similar depths during both expeditions (330–476 m), but DO concentrations were slightly lower during *Tara Oceans* (1.3–4.4 µmol kg^−1^) than in our study (5.2–10.6 µmol kg^−1^). This difference may reflect reduced ventilation associated with the decaying 2011 event, compared with enhanced lateral oxygen transport together with stronger upwelling during the persistent La Niña conditions in 2022. In both cases, OMZ core waters were identified as ESSW (Fig. 1), indicating comparable deep-water characteristics and microbial habitat conditions (61).

### Water column variability on microbial composition

**Fig 2:**
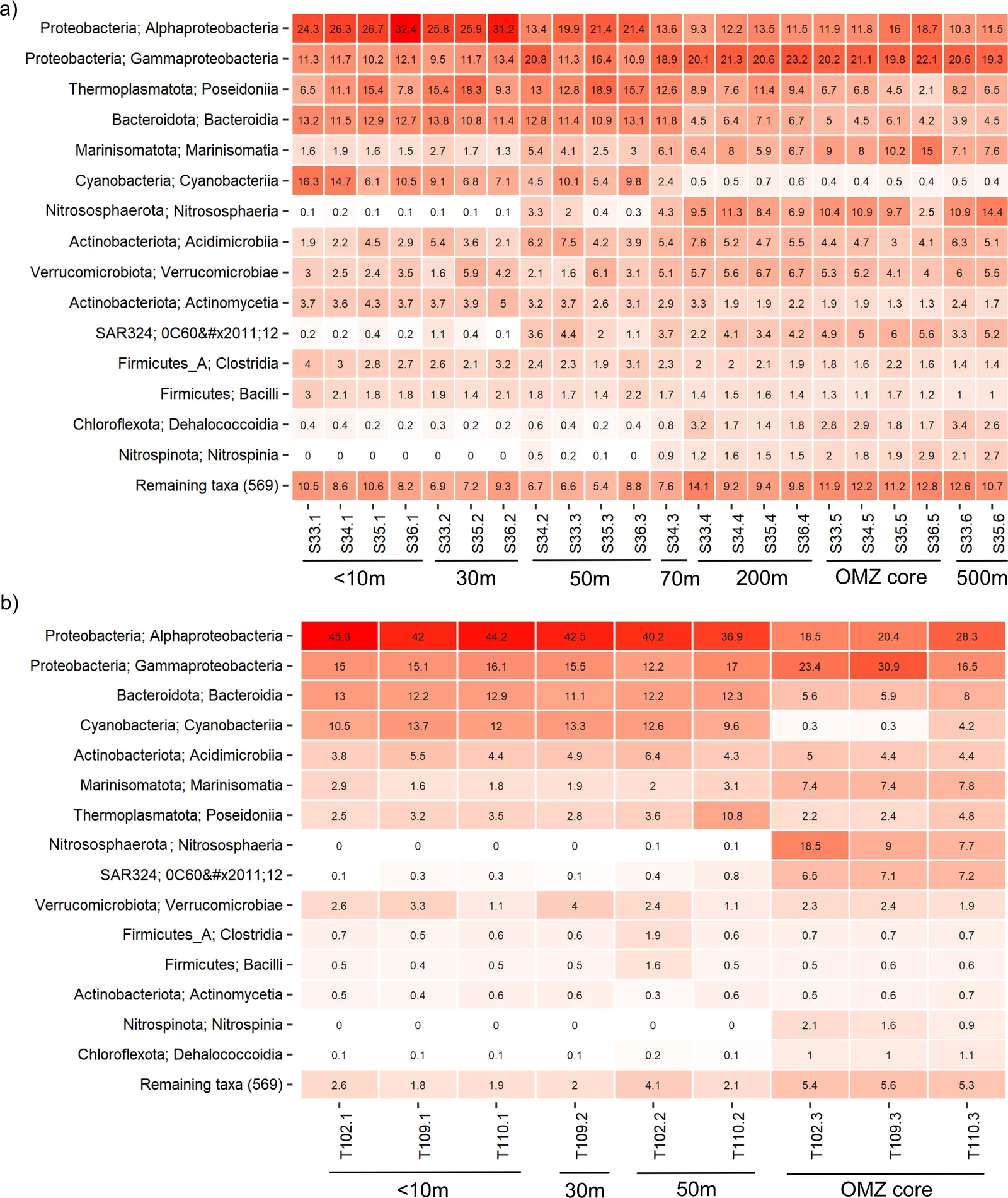
Relative abundance of microbial classes based on various depth niches in the ETP collected during SO287 cruise (**a**) and *Tara Oceans* projects (**b**).

The microbial community observed in this study showed a vertical distribution pattern with characteristic microbes for each sampling depth and was thus comparable to the results from other OMZs (3, 63). Within our 22 metagenome data sets, an average of 34.82% of clean raw reads could be assigned to microbes (Table S2). The majority of assigned reads were bacteria (79.1%–94.7%), while the rest were assigned to archaea (5.3%–20.9%). Most dominant groups clearly differed in abundance between the euphotic layer (<10–70 m) and deeper waters (70–500 m) (Fig. 2a; Fig. S5a). Alphaproteobacteria and Gammaproteobacteria were the top abundant groups across all the depths, accounting for 9.3%–32.4% and 9.5%–23.2%, respectively (Fig. 2a). In the euphotic layer, dominant groups were Poseidoniia (6.5%–18.9%), Bacteroidia (10.8%–13.2%), and Cyanobacteria (4.5%–16.3%), while in deeper waters, microbial communities were dominated by Nitrososphaeria (2.5%–14.4%) and Marinisomatia (5.9%–15%) (Fig. 2a). Some other groups also showed higher abundance in deeper waters compared with the euphotic layer, e.g., Dehalococcoidia and Nitrospinia. The highest abundance of Marinisomatia (15%) and a relatively lower abundance of Nitrososphaeria (2.5%) were observed in the OMZ core of S36, which was also characterized by the lowest DO concentration in our entire data set (5 μmol kg^−1^) (Fig. 2a). In the 50-m samples from stations S33 and S34, the dominant groups were mixed with groups from the euphotic zone (e.g., Cyanobiaceae, Flabacteriaceae) and deeper waters (e.g., Nitrosopumilaceae, Thioglobaceae) (Fig. S5a). This pattern is consistent with enhanced upwelling, introducing nutrient-rich, low-oxygen waters into the lower euphotic zone and allowing the co-occurrence of surface- and deep-water taxa. This might be due to both low DO and high nutrient concentrations (NO_3_^−^, PO_4_^3−^) in those two samples, which might be beneficial for both deep water and surface water microbes. The vertical distribution of microbial communities can be caused by a combination of many variables across the water column, including temperature, DO, nutrients, and light (63). We found that DO, temperature, NO_3_^−^, and PO_4_^3−^ could explain a larger proportion of microbial composition structure, respectively (~49.7%–50.5%; Table 1), compared with other factors, e.g., NO_2_^−^, salinity (~0.21%–0.28%). The explanatory power of DO, temperature, and nutrients speaks for ocean deoxygenation as a key factor that determines the ETP microbial community, as deoxygenation is largely scaled with nutrient supply, and DO decreases in parallel with temperature in OMZ waters (30, 64).

**TABLE 1:** BIO-ENV outputs show the proportion of the microbial community pattern or microbe-mediated nitrogen metabolism from SO287 data sets that can be explained by each variable or multiple variables^*a*^

Variable	Proportion (*R*^2^)	
	Microbial community	Nitrogen metabolism
Single variable		
NO_3_^−^	0.505	0.387
Temperature	0.499	0.578
DO	0.498	0.627
PO_4_^3−^	0.497	0.525
SiO_2_	0.465	0.525
Turbidity	0.459	0.532
Depth	0.457	0.616
Salinity	0.278	0.225
N:P	0.289	0.205
NO_2_^−^	0.207	0.247
Multiple variables		
DO + NO_2_^−^	0.630	0.673
DO + temperature + depth + NO_2_^−^	0.722	0.869
DO + depth + NO_2_^−^ + NO_3_^−^ + turbidity	0.752	0.882
DO + temperature + depth + NO_2_^−^ + NO_3_^−^ + turbidity + salinity	0.794	0.91

^*a*^ All individual environmental variables showed significant relationships with microbial community patterns based on permutation tests (*P* = 0.001). Multivariable BIO-ENV models explained a higher proportion of variation than single variables and were selected based on AIC optimization.

Low DO concentration is the main indicator directly associated with ocean deoxygenation (3, 30). Abundant microbial groups, including Alphaproteobacteria (Pelagibacteraceae (SAR11), Rhodobacteraceae), Bacteroidia (Flavobacteriaceae), Cyanobacteriia, Poseidoniaceae, D2472 (SAR86), decrease significantly when DO declines below ~80 µmol kg^−1^ (Fig. S6 and S7), suggesting that those microbes would be less abundant when OMZs intensify or expand. In contrast, Gammaproteobacteria (Thioglobaceae (SUP05), UBA868), Dehalococcoidia, Nitrospinia, Marinisomatota (TCS55, UBA1611), Nitrososphaeria, one SAR324 class, Acidimicrobiia, and UBA1096 (Verrucomicrobiota), increased dramatically when DO drops below 120 or 80 μmol kg^−1^, suggesting an advantage for those groups under OMZ expansion. Some of those groups have been found dominant in different DO regimes in other OMZ regions (4). For example, SUP05 and SAR324 dominated in OMZ waters (DO < 70 µmol kg^−1^), while Flavobacteriaceae, SAR11, and SAR86 dominated in waters with DO >70 µmol kg^−1^ in the ETSP off central Chile (64, 65). Nitrospinia, SAR324, and Marinisomatia have been found more abundant in the OMZ core, while Synechococcales, Rhodobacterales, SAR86, Flavobacterales, and Verrucomicrobiales were found to be more abundant in the euphotic layer of OMZs in the Gulf of Alaska and the ETNP off Mexico (63, 66). Our findings are thus well in line with previous studies and highlight that a DO concentration of about 80 μmol kg^−1^ can be a threshold for a shift in microbial community composition in the ETP.

Furthermore, we found that the microbial composition present at a DO range between 5 and 20 μmol kg^−1^ and between 20 and 60 μmol kg^−1^ were clustered together, as shown in the PCoA plot (Fig. S8), suggesting that the present microbes are at least somewhat tolerant to this range of DO concentrations. The sample with the lowest DO concentration (5 μmol kg^−1^) was, however, quite different from the other hypoxic water samples (Fig. S8), with a substantially higher abundance of Marinisomatia and a relatively lower abundance of Nitrososphaeria (Fig. 2a). Nitrososphaeria has also been found in lower abundances within anoxic waters compared with other OMZ waters in the ETNP (10), while Marinisomatota has been found to prefer low DO habitats in various OMZs (30, 66, 67). The sensitivity of individual microbial groups to DO concentrations is key for understanding the microbial community response to deoxygenation.

### Oxygen gradients shaping microbial alpha diversity and abundance

**Fig 3:**
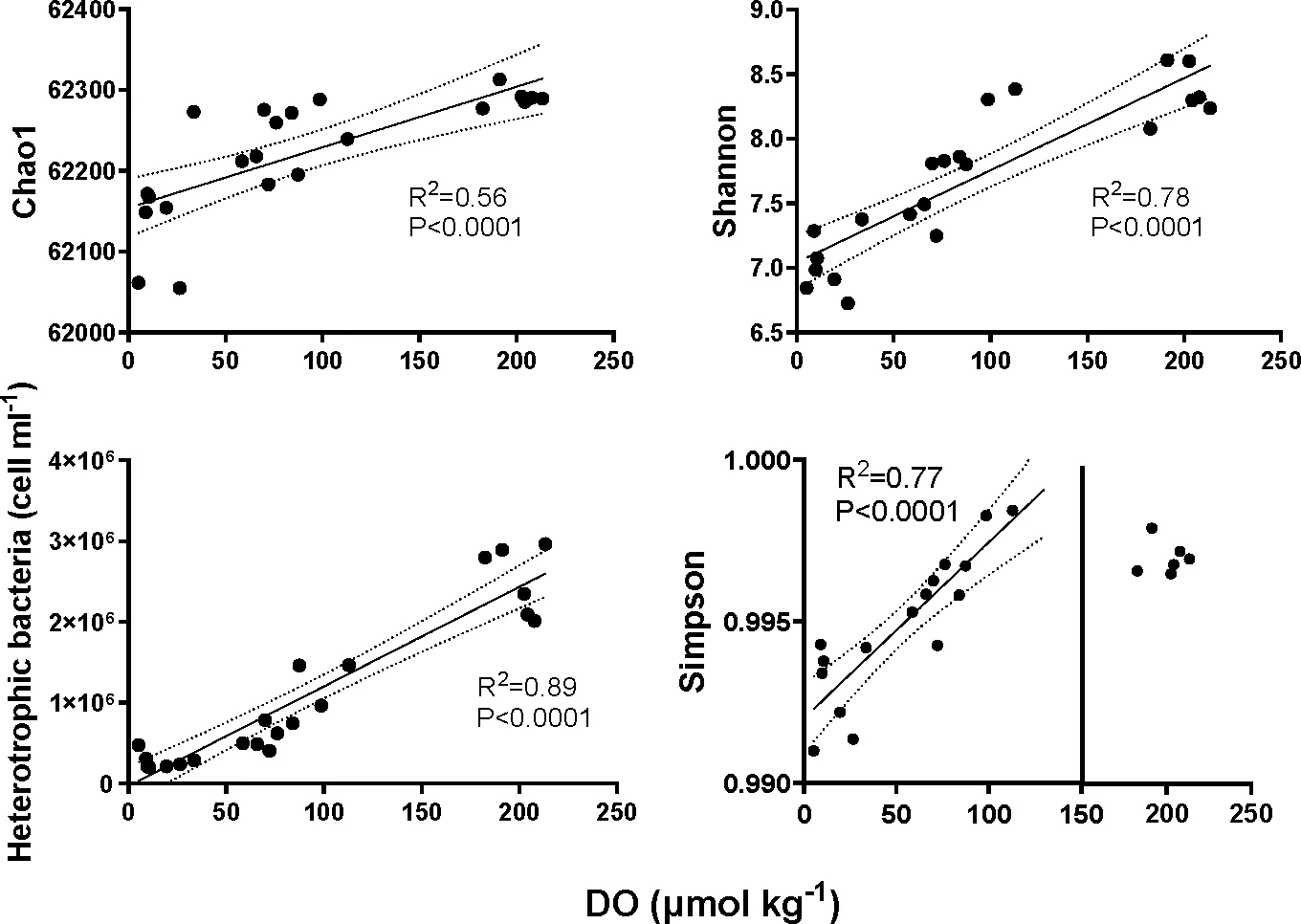
The correlation between DO and alpha diversity of microbial community (Chao1, Shannon, Simpson) and abundance of heterotrophic bacteria (cell counts) with all 22 samples from SO287 data sets.

DO was determined as a key factor affecting not only microbial composition but also microbial diversity in many oceanic OMZs (30, 68–70). Within our own data sets, all three indices of microbial diversity (Chao1, Shannon, and Simpson) were positively correlated with DO in a range of 5–210 µmol kg^−1^ in the ETP OMZ (Fig. 3; *R*^2^ = 0.56, 0.78, and 0.77, *P* < 0.0001). Moreover, the abundance (cell counts) of heterotrophic prokaryotes was also positively correlated with DO (Fig. 3; *R*^2^ = 0.89, *P* < 0.0001). Thus, our data suggest that the decline of oxygen would lead to a loss of microbial total abundance and diversity in the ETP. A possible explanation would be that there has been a decline of available ecological niches in the OMZ compared with well-oxygenated waters, which would be more significant than the increase in niches resulting from electron acceptor partitioning (71). The decrease in niches in the OMZ could be relevant with reduced energy availability due to factors, such as absence of solar radiation, less easily accessible organic material, and lower redox potentials (71).

In other oceanic OMZ systems, alpha diversity has predominantly been assessed using 16S rRNA amplicon sequencing, which typically yields lower richness and diversity estimates than shotgun metagenomic approaches (7, 63, 72). Reported Chao1 values in these studies generally range from hundreds to a few thousand taxa (7, 63, 72), substantially lower than the values observed here (>60,000; Table S5). Similarly, Shannon diversity indices reported for OMZ microbial communities in regions such as the BoB and the Arabian Sea are typically smaller than 5 (7, 72), whereas values from the Mexican OMZ range 4–8 (63). In comparison, the Shannon indices in our study (6.7–8.6) fall within the upper range of previously reported values but are consistently higher than most 16S-based estimates. This discrepancy is expected, as shotgun metagenomic sequencing combined with species-level classification captures a broader range of taxa, including rare members, leading to elevated diversity estimates (73). Therefore, absolute Shannon values are not directly comparable across methodological frameworks across various studies on OMZ microbial diversity.

### Oxygen gradients shaping microbial nitrogen metabolisms

Our sampling spans a broad oxygen range from oxic to suboxic conditions, remaining above fully anoxic thresholds typical of oxygen-depleted zones (ODZ) cores. Consistent with this, the absence of SNM and the persistence of relatively high NO_3_^−^ concentrations in the OMZ cores indicate efficient nitrite consumption and limit net nitrogen loss (16). This contrasts with anoxic systems such as the ETSP, the ETNP, and the Arabian Sea, where strong SNMs, NO_3_^−^ depletion, and high N_2_ production reflect the dominance of anaerobic nitrogen loss pathways by denitrification and anammox (11, 12, 74).

**Fig 4:**
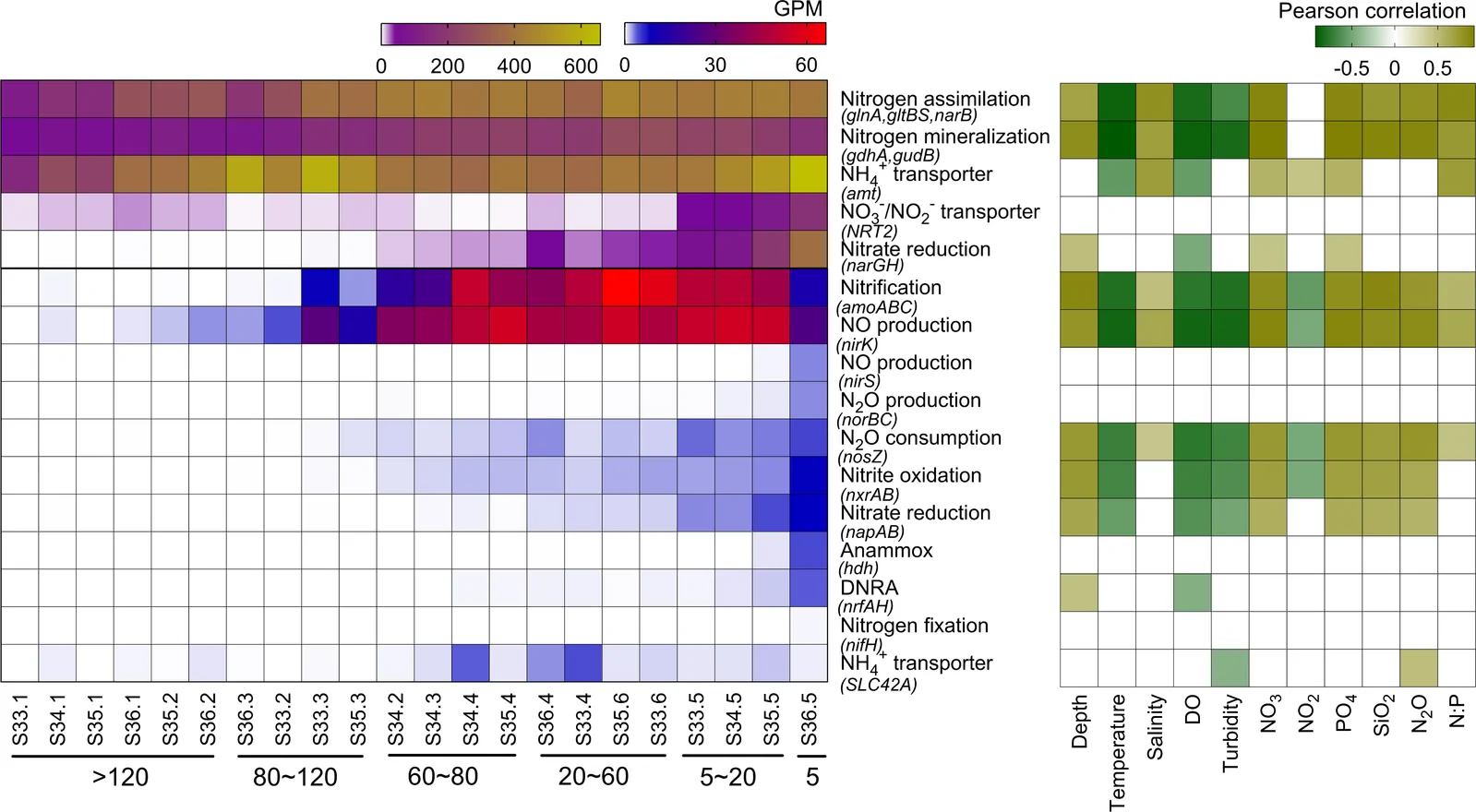
Genes per million (GPM) of each nitrogen metabolism in the metagenome data sets from SO287 samples, and their correlations with physiochemical parameters (*P* < 0.05). In the left panel, for the first five rows, a coarser range (0–600) with the left color bar was used, whereas the rest of the rows, a narrower range (0–60) with the right color bar was used.

**Fig 5:**
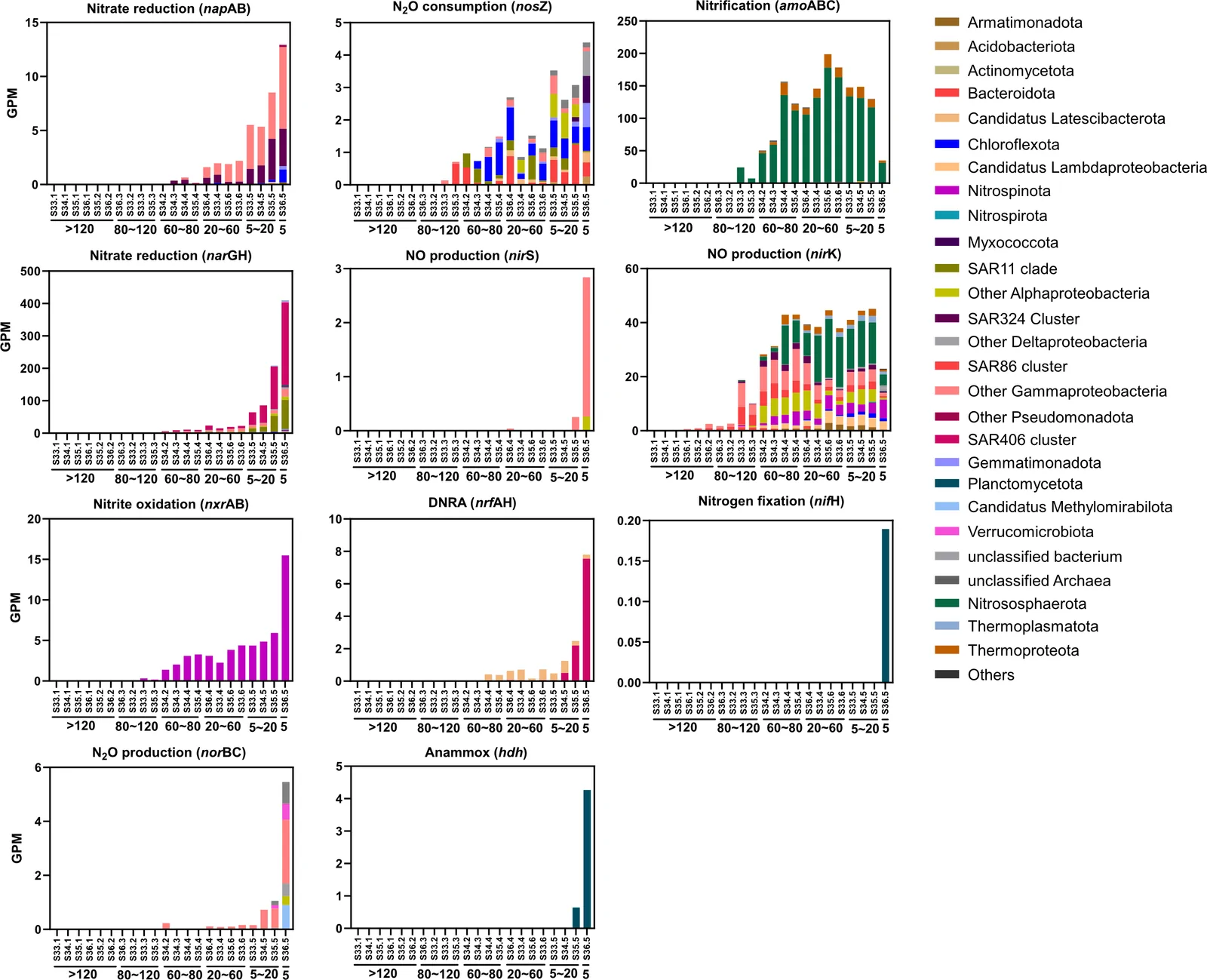
Microbial taxa of the genes involved in nitrogen cycling and their GPM value in each sample from SO287 data sets with the range of DO concentrations.

Across this low-oxygen (but not anoxic) gradient, nitrogen transformation pathways exhibit distinct oxygen preferences, highlighting a complex and dynamic gene capacity for the nitrogen cycle compared with fully oxygenated waters (6). Genes associated with core nitrogen processes, including nitrogen assimilation, nitrogen mineralization, NH_4_^+^ transporter (*amt*), nitrate reduction (*nar*GH, *nap*AB), nitrite oxidation (*nxr*AB), nitrification (*amo*ABC), NO production (*nir*K), N_2_O consumption, and DNRA, showed a negative correlation with DO (Fig. 4). While genes linked to some pathways (e.g., nitrogen assimilation, nitrogen mineralization, NH_4_^+^ transporter (*amt*)) persist across all oxygen regimes, others display clear niche partitioning (Fig. 4). For example, genes encoding nitrification (*amo*ABC) were mostly present in low-oxygenated waters (DO at 80–120 µmol kg^−1^) and prevailed in hypoxic waters (DO at 5–80 µmol kg^−1^) but declined in suboxic waters (DO ~5 µmol kg^−1^) (Fig. 4), which might be explainable by their need for oxygen. In contrast, genes associated with nitrate reduction (*nar*GH, *nap*AB) were significantly more prevalent in the OMZ core (<20 µmol kg^−1^), exhibiting particularly high relative abundances in suboxic waters. Nitrate reduction (*nar*GH) was positively correlated with NO_3_^−^ concentration (Fig. 4), which serves as the next favorable electron acceptor after oxygen in the OMZ core (8). DNRA-associated genes (*nrf*AH), which mediate nitrate reduction to ammonium and thus represent a nitrogen-retention pathway competing with nitrogen-loss pathways, showed a similar distribution pattern, with elevated relative abundances in the OMZ core and a maximum in suboxic waters (Fig. 5). Its co-occurrence with anammox-associated genes (*hdh*) suggests a potential coupling that may recycle ammonia to fuel anammox, as previously observed in the ETSP and Arabian OMZs (12, 75).

In suboxic waters (DO ~5 µmol kg^−1^), genes associated with canonical denitrification (*nar*GH, *nir*S, *nor*BC, *nos*Z), anammox (*hdh*), and DNRA (*nrf*AH) all reach peak abundances (Fig. 4 and 5), indicating an intensification of nitrogen loss potential. Genes encoding nitrite oxidation (*nxr*AB) also peak at suboxic waters (DO ~5 µmol kg^−1^), increasing from low-oxygenated waters (DO at 80–120 µmol kg^−1^) into hypoxic waters (5–60 μmol kg^−1^) (Fig. 4). Previous observations from the ETNP reported that relative gene abundances of nitrite oxidation and nitrate reduction (*nar*GH, *nxr*AB) were low at hypoxic (DO at 20 μmol kg^−1^) but increased sharply in SNM in the ETNP (76). These results suggest that the peak abundance of these gene capacities shifts across oxygen gradients, occurring in SNM conditions in the ETNP but in suboxic waters in our system. The lack of a SNM, despite the coincident enrichment of genes mediating both NO_2_^−^ production (nitrate reduction) and NO_2_^−^ consumption (nitrite oxidation and nitrite-reducing denitrification) in suboxic waters, suggests that oxygen concentrations (DO ~5 µmol kg^−1^) remain sufficient to support nitrite-oxidizing bacteria. As proposed by Sun (16), oxygen limitation of nitrite oxidizers is a critical driver of nitrite accumulation in anoxic waters. In our low-oxygen waters (DO ≥ 5 µmol kg^−1^), nitrite oxidizers may outcompete nitrite-reducing denitrification processes, probably limiting NO_2_⁻ buildup (16).

Together, these results highlight that our study captures a transitional low-oxygen regime, distinct from the anoxic OMZ cores where much previous work has focused (17, 76, 77). Rather than an anaerobic system dominated by nitrogen loss capacity, we identify a low-oxygen niche where the genetic potential for both oxidative and reductive nitrogen cycling coexists: genes for ammonia oxidation (*amo*ABC), nitrite oxidation (*nxr*AB), and nitrate reduction (*nar*GH) are all abundant in waters with DO of 5–80 µmol kg^−1^ (Fig. 4). This co-occurrence is consistent with observations from other oceanic OMZs. For example, genes encoding *amo*A, *nxr*B, and *nar*GZ prevailed at 500 m where DO was 5–20 µmol kg^−1^ in the BoB OMZ (7), while *amo*, *nxr*, and *nar* genes co-occurred in the OMZ core (DO < 25 µmol kg^−1^) off Namibia, although these genes were also detected at 10 m where DO exceeded 100 μmol kg^−1^, possibly reflecting active upwelling in this shallow system (<400 m deep) (32). In our system, the decline in nitrification (*amo*ABC) and increase in denitrification and anammox potential at ~5 µmol kg^−1^ are also consistent with patterns observed in the northern BoB OMZ, although the transition occurred at lower DO (<2.5 µmol kg^−1^) there (78). The oxygen-dependent partitioning of nitrogen-cycling potential has important implications for predicting nitrogen-cycle responses to future deoxygenation scenarios: if the OMZ core (5–20 μmol kg⁻¹) contracts while low-oxygenated waters (DO < 120 µmol kg^−1^) expand as predicted, the relative distribution of oxidative and reductive nitrogen-cycling potentials may shift, with consequences for the balance among nitrogen transformation pathways and associated nitrogen loss.

**Fig 6:**
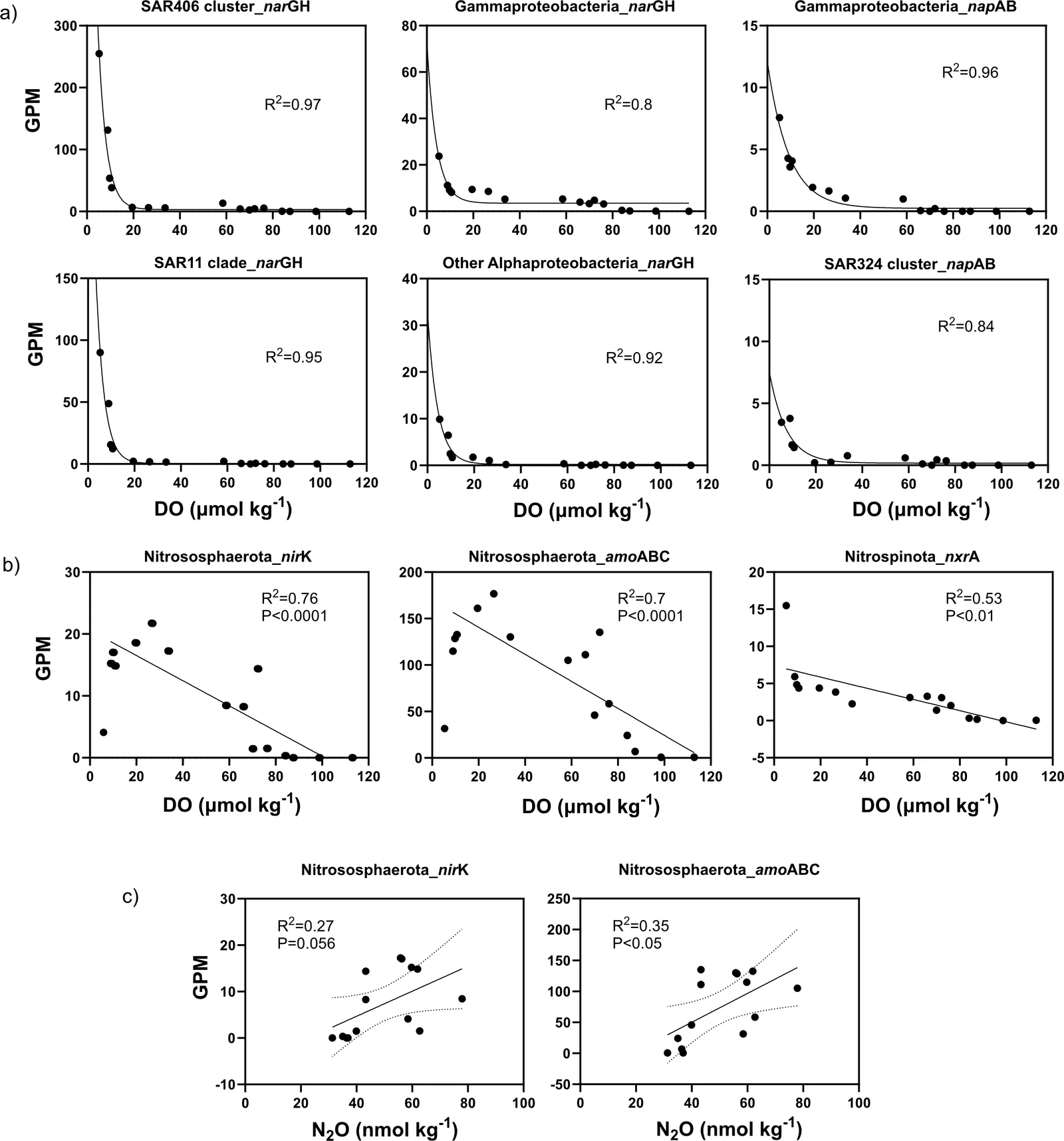
Relationships between environmental parameters and nitrogen cycling gene abundance in microbial taxa from SO287 data sets. (**a)** Exponential correlations between DO concentration and the GPM value of dominant microbial taxa involved in dissimilatory nitrate reduction to nitrite (*narGH* and *napAB* genes); (**b**) linear correlations between DO concentration and the GPM value of some dominant microbial taxa involved in key nitrogen transformation pathways (*nirK*, *amoABC*, and *nxrA* genes); (**c**) linear correlations between N_2_O concentration and the GPM value of Nitrososphaerota *nir*K and *amo*ABC genes. Only samples (DO < 120 µmol kg^−1^) collected by our sampling were considered. The dashed line indicates the 95% confidence interval.

Nitrification and denitrification are two main processes that contribute to N_2_O production (79). Here, we found that both genes encoding nitrification (*amo*ABC) and NO production (*nir*K) were positively correlated with N_2_O concentrations in the investigated waters (*R* = 0.76, 0.83 respectively) (Fig. 4; Table S4). Nitrososphaerota were the major ammonia oxidizers with the potential to mediate nitrification, and the dominant group for potential NO production via *nir*K (Fig. 5). Both Nitrososphaerota-encoded *nir*K and *amo*ABC were positively correlated with N_2_O concentration (*R*^2^ = 0.27, *P* = 0.056; *R*^2^ = 0.35, *P* < 0.05, respectively) but negatively correlated with DO (*R*^2^ = 0.76, 0.7, respectively, *P* < 0.0001) (Fig. 6b and c), suggesting that deoxygenation may increase the potential contribution of AOA to N_2_O production. The contribution of AOA to N_2_O production in oxygenated and OMZ waters has been highlighted in previous studies (14, 80, 81), and yet its precise mechanism remains unclear. Recent studies proposed hybrid N_2_O formation by AOA via the coupling of precursor substrates, NH_2_OH (via *amo*ABC) and NO (via *nir*K) (80, 82), to be a significant source for N_2_O production in ETNP OMZs (DO < 30 µmol kg^−1^) (80).

During our sampling campaign, in suboxic waters (~5 µmol kg^−1^), the relative abundance of genes encoding canonical denitrification (*nir*S, *nor*BC) was substantially higher than Nitrososphaerota encoding nitrification (*amo*ABC, *nir*K), suggesting greater genetic potential for N_2_O production via denitrification relative to nitrification (Fig. 5). This pattern aligns with previous studies reporting substantially higher N_2_O production rates from denitrification compared with nitrification in suboxic waters (81, 83, 84), though we emphasize that gene abundances indicate metabolic potential rather than measured activity. In broader OMZ core waters (5–20 μmol kg^−1^), where denitrification marker genes (*nar*GH, *nor*BC, *nir*S) were higher than waters at DO > 20 µmol kg^−1^ and nitrification-associated genes (*amo*ABC) were found significantly higher than in suboxic water (Fig. 5), indicating the co-occurrence of genetic markers for multiple N₂O production pathways. Such co-occurrence may partly reflect microscale redox heterogeneity associated with organic matter particles, where anoxic microenvironments can support denitrification under higher ambient oxygen conditions (85, 86), although particle size distributions were not assessed in this study. Furthermore, the last step of denitrification, N_2_O consumption encoded by *nos*Z, exhibited the highest relative abundance in suboxic waters (Fig. 4 and 5), indicating an increased genetic potential for N₂O reduction in these waters, which may help explain the relatively lower N_2_O concentrations observed compared with adjacent waters (Table S1) (87, 88). Furthermore, the genetic potential for N_2_O consumption (*nos*Z) was not only negatively correlated with DO but also positively correlated with N_2_O concentrations (Fig. 4), as previously observed in the ETSP OMZs (22). The occurrence of N_2_O consumers started in low-oxygen water (Fig. 4 and 5), supporting the recent findings that the potential of N_2_O consumption may occur in slightly to fully oxic waters instead of only in anoxic environments (21, 87). This increases the possibility that part of N_2_O induced by nitrification could potentially be removed by N_2_O consumers even in oxygenated waters (89).

The composition of taxonomic carriers was different within different steps involved in canonical denitrification (*nar*GH, *nir*S, *nor*BC, *nos*Z) (Fig. 5), consistent with the concept of “modular denitrification” where distinct microbial populations perform subsets of the denitrification pathway rather than the complete sequence within single organisms (84). This metabolic partitioning may facilitate niche differentiation and coexistence of diverse denitrifier communities across oxygen gradients. For nitrate reduction (*nar*GH), the dominant groups were Marinimicrobia, followed by the SAR11 clade and less abundant groups of Gammaproteobacteria and other Alphaproteobacteria (Fig. 5). Moreover, the relative abundance (GPM) of *nar*GH encoded by each of aforementioned groups were highly negatively correlated with DO (*R*^2^ >0.8; Fig. 6a and b). Our study strengthens the potential key role of the SAR11 clade for nitrate reduction in OMZs, which might be reinforced with decreasing DO, as previously suggested for the ETNP and BoB (7, 90). Gammaproteobacteria were the main denitrifier encoding *nir*S and *nor*BC, particularly in suboxic waters (Fig. 5). Together with their dominance among *nar*GH-encoding taxa, we propose Gammaproteobacteria to be the key potential denitrifiers in the ETP OMZ core. The functional groups carrying the potential for N_2_O consumption were quite diverse across all DO concentrations, including groups affiliated with SAR11 and other Alphaproteobacteria, Bacteroidota, Gemmatimonadota, Gammaproteobacteria, and Chloroflexota (Fig. 5). Many of those groups have been detected also in other OMZs (89), e.g., denitrifying Alphaproteobacteria were found to express *nos*Z in oxic and suboxic waters in the Arabian Sea (21). Not much difference in habitats was observed among various N_2_O consumers.

Marinimicrobia was the dominant group for the process of DNRA in the OMZ core, while in low oxygenated and hypoxic waters (20–80 μmol kg^−1^), Candidatus Latescibacterota was the key group (Fig. 5). Marinimicrobia has been previously found to be the only abundant DNRA group in OMZs (17). Nitrospina dominated nitrite oxidation (*nxr*AB) in our samples (Fig. 5), as observed in other OMZs including the ETSP and ETNP (77, 91), and its abundance increased significantly toward lower DO concentrations (*R*^2^ = 0.53, *P* < 0.01; Fig. 6b). Planctomycetota was the only group with genetic capacity for anammox and nitrogen fixation in the OMZ core (Fig. 5). Except for AOA, several bacteria also encoded *nir*K across a broad DO range (<120 µmol kg^−1^), including the SAR11 clade and other Alphaproteobacteria, the SAR86 cluster and other Gammaproteobacteria, as well as Nitrospinota. A previous study identified a similar niche of *nir*K-carrying organisms using MAGs, which showed a maximum of nitrite reducers (*nir*K) at the oxycline and upper OMZ, with the most abundant MAGs affiliated with Nitrospina and AOA (17). Overall, when considering both the relative abundance of key nitrogen metabolisms and their functional microbial groups along different waters (Fig. 5), DO is the most significant factor (*R*^2^ = 0.627) compared with other variables (e.g., nutrients, temperature) (Table 1). This highlights the significance of DO not only connecting to nitrogen cycling (8, 12), but also in the niche partitioning of their functional groups.

### Comparing microbial composition and diversity under two La Niña years

Although deoxygenation was determined as a key factor affecting not only microbial composition but also microbial diversity in many oceanic OMZs (30, 68–70), how microbial composition and diversity change over long-term effects under comparable ENSO phases has not been understood yet. As direct metagenomic comparisons of these two data sets with neutral or El Niño conditions in the ETP are currently unavailable, here we interpret the 2011–2022 comparison as a comparison between two La Niña states rather than as a direct test of ENSO effects on microbiome and their functional potential. Because both sampling efforts were conducted during La Niña conditions, large basin-scale environmental differences were not expected. Consistent with this expectation, the *Tara Oceans* (2011) and SO287 (2022) data sets were broadly comparable, showing similar numbers of observed species (>60,000) and comparable sequencing saturation (Table S5; Fig. S9), despite differences in sequencing depths and percentage of taxonomic assigned reads (Table S2). The clades identified in the *Tara Oceans* data set and our samples showed similar distributions along vertical profiles. For example, SAR11 (Pelagibacteraceae) was dominant in both data sets but turned out to be more represented in the *Tara Oceans* data set (12.9%–27%) than in SO287 (4.3%–16.2%) (Fig. S5). In contrast, Posidoniia (belonging to Thermoplasmatota) showed higher representation in SO287 (average 10.4%) compared with *Tara Oceans* (average 4.0%) (Fig. 2). Moreover, Flavobacteriaceae, Rhodobacteraceae, and D2472 were more represented in the Tara euphotic layer (<50 m) than in SO287, while Nitrosophaeria and one SAR324 class showed an opposite trend (Fig. 2; Fig. S5). These differences could indicate modest temporal variation in community composition between the two sampling years; however, environmental variability and methodological differences limit direct quantitative comparison.

Alpha diversity indices further supported overall similarity between the two La Niña years. Comparing the alpha diversity between the *Tara Oceans* data set and the SO287 data set for either before or after rarefaction of reads, we only observed a higher Shannon index in the surface (<10 m) samples of our data sets (Fig. S10 and S11) and no significant difference for Simpson indices in all samples. Chao1 was higher in our data set than the *Tara Oceans* data set when the reads were rarefied, while there was no difference when reads were not rarefied. This is because the sequencing depths of the *Tara Oceans* samples were quite diverse, and the rarefaction of reads decreased the number of Chao1 (Table S5). Taken together, these indices indicate broadly comparable microbial diversity between SO287 and *Tara Oceans* data sets in both upper layers (<50 m) and OMZ core.

Altogether, we did not observe large shifts in microbial composition and diversity when comparing the La Niña years of 2011 and 2022. Studies found that El Niño events would deepen the OMZ upper boundary and decrease the OMZ volume (37), which then would expand the habitat of microbes that prefer higher DO concentration. However, only limited studies have explored the possible effect of ENSO events on the microbial community in the ETP (39, 92). SAR11, Flavobacteriaceae, and Rhodobacteraceae were detected as less abundant during El Niño in 2015 compared with La Niña in 2016 in surface waters (5–10 m) around the Galápagos Archipelago (islands located in the west of our sampling sites) (92). Thermoplasmatota were more abundant during La Niña in 2018 compared with the El Niño period in 2019 in the ETNP OMZ (39). Those groups prefer higher DO concentration, and the decline of their relative abundance during the El Niño phase might be due to an increase in cyanobacteria, especially Synechecoccus (39, 92). In this study, we found a higher relative abundance of Thermoplasmatota and lower relative abundances of SAR11, Flavobacteriaceae, and Rhodobacteraceae, but similar abundances of cyanobacteria in 2022 compared with 2011 under similar La Niña conditions (Fig. S5). This might speak for the distinct resilience of those groups towards ENSO events; for example, Thermoplasmatota might recover rapidly after El Niño declines, while other groups (SAR11, Flavobacteriaceae, and Rhodobacteraceae) do not have such resilience.

### Comparing microbial nitrogen metabolisms between two La Niña years

**Fig 7:**
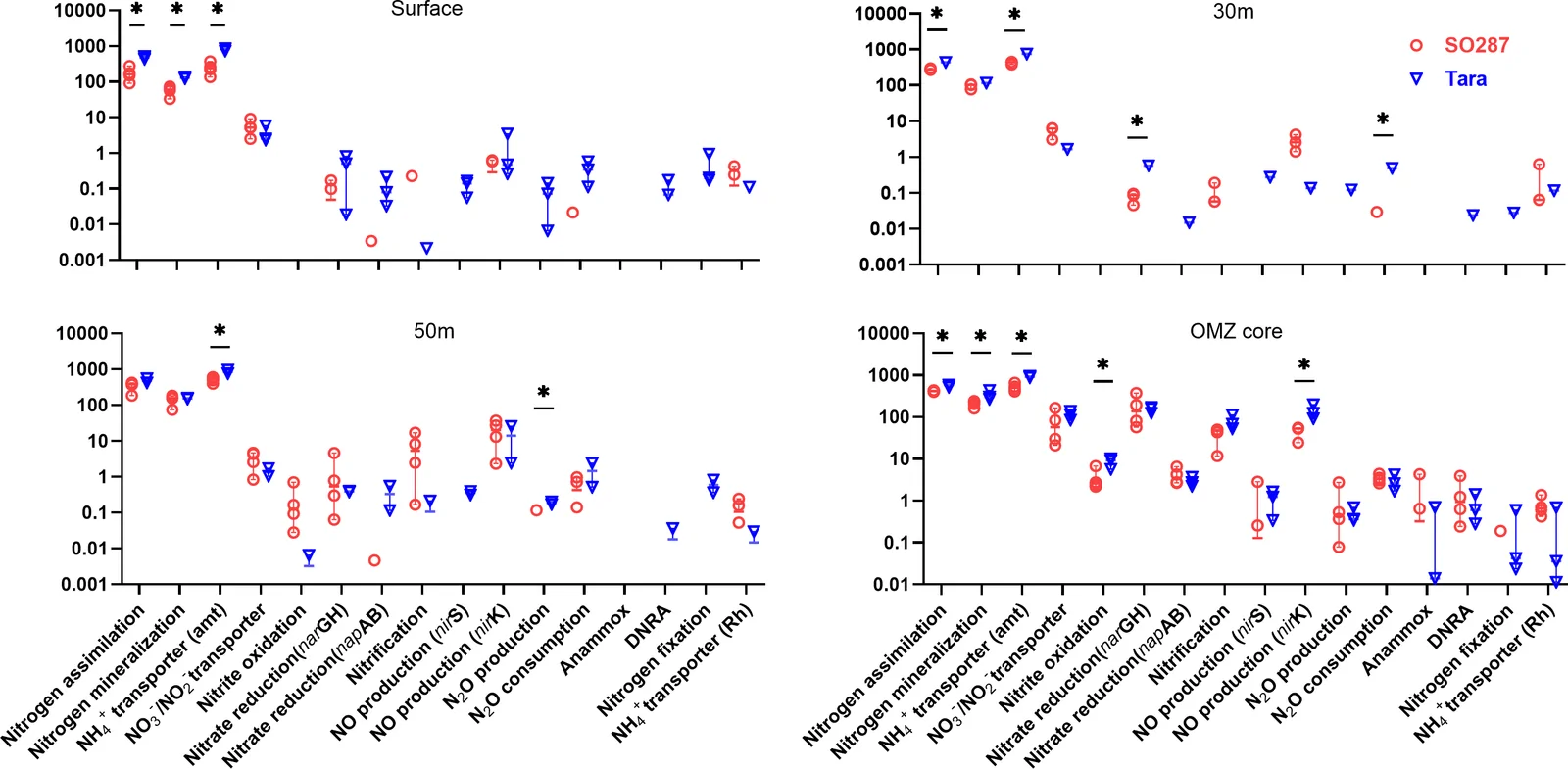
Comparison of relative abundance (genes per million, GPM value) of each nitrogen metabolism pathway between *Tara Oceans* and SO287 data sets at four depths. Asterisks indicate statistically significant differences (*P* < 0.05). At the surface, 30 m, and 50 m depths, several nitrogen transformation pathways (NO production via *nir*S, DNRA, nitrogen fixation) were only detected in the *Tara Oceans* data sets.

**Fig 8:**
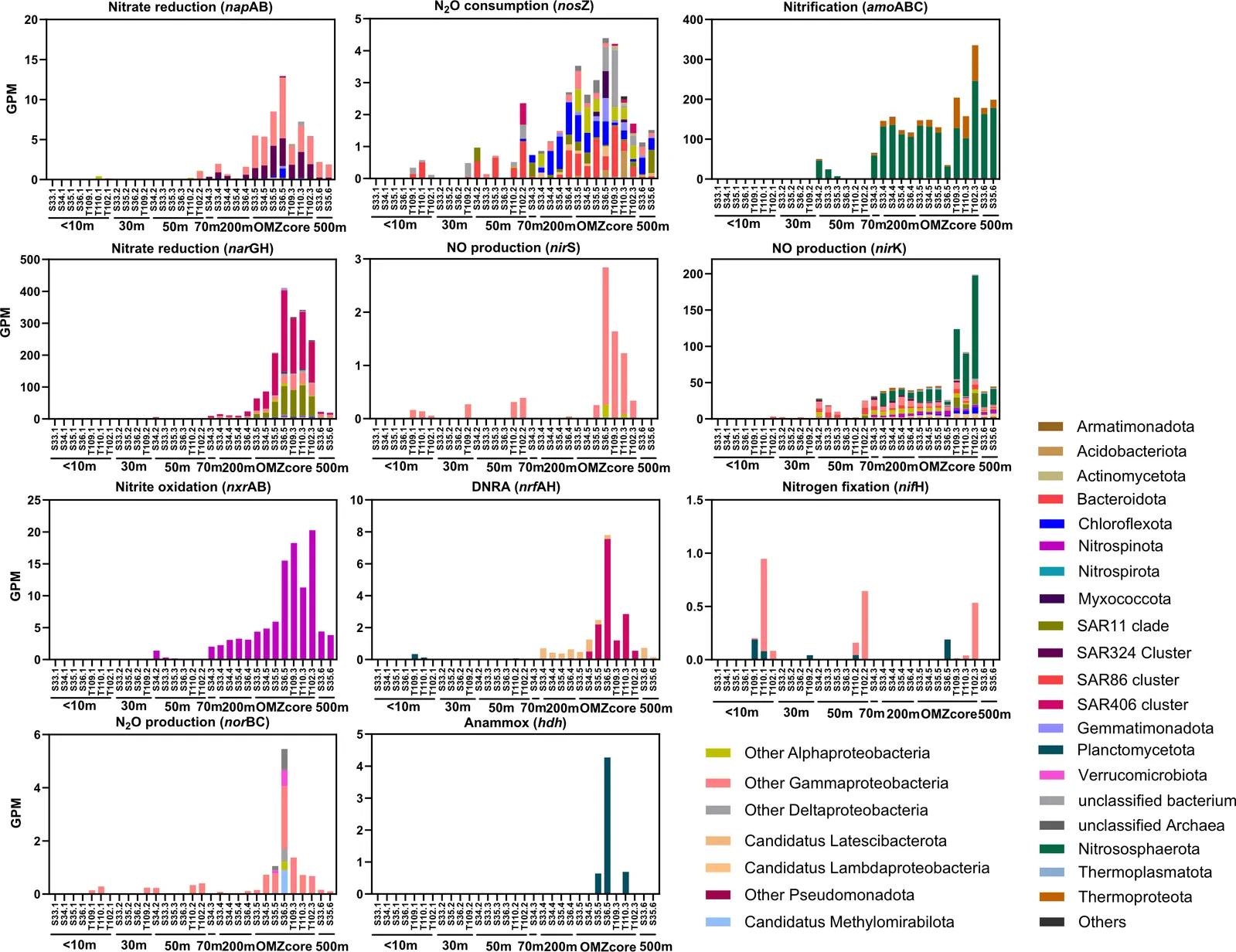
Microbial taxa of the genes involved in nitrogen cycles and their GPM value in each sample from both *Tara Oceans* and SO287 data sets.

We compared the microbial potential for nitrogen cycle processes between two La Niña years using the *Tara Oceans* (2011) and SO287 data sets (2022) from four different water depths (<10 m, 30 m, 50 m, OMZ core) (Fig. 7 and 8). Given that both years represented the same ENSO phase, only modest differences in nitrogen cycling genes were expected. Accordingly, the overall composition of nitrogen functional groups showed broadly comparable patterns between years, and most differences were subtle rather than indicative of major shifts in nitrogen cycling potential. Several pathways were found with higher relative abundance in the *Tara Oceans* samples compared with ours (Fig. 7). For example, within the OMZ core, gene abundance for nitrite oxidation and NO production (*nir*K) were significantly higher in *Tara Oceans* samples than in ours (Fig. 7). However, unlike our suboxic sample, none of the *Tara Oceans* OMZ core samples showed a similar potential for canonical denitrification (N_2_O production (*nor*BC) and NO production (*nir*S)), anammox (*hdh*), or DNRA (Fig. 8), although *Tara Oceans* OMZ core samples were also taken from suboxic waters and should thus be comparable (Table S1, DO<5 µmol kg^−1^). This difference might result from other factors, for instance organic matter availability and quality in the OMZ core, as organic matter is a limiting factor for denitrification (79, 93), and it can provide essential substrates, nitrite, and ammonium, for anammox (94). Moreover, in oxic samples derived from the <10 m, 30 m, or 50 m, canonical denitrification, N_2_O consumption (*nos*Z), N_2_O production (*nor*BC), and NO production (*nir*S), were found in higher relative abundances in *Tara Oceans* samples (Fig. 7 and 8). Possible explanations might be a difference in particle microenvironments between both cruises (20, 95) or the presence of aerobic denitrifiers (96) during *Tara Oceans* sampling in the euphotic layer. Because *Tara Oceans* targeted the 0.2–3-µm fraction, whereas our samples included all cells >0.22 µm, particle-associated anaerobic niches may partly explain these differences, especially in OMZ core samples; however, the elevated denitrification genes in *Tara* euphotic samples indicate that particle size alone cannot account for the observed patterns. Altogether, our analysis suggests a higher potential for nitrogen loss in suboxic waters in 2022 compared with 2011, while in the euphotic zone, the opposite could be found.

There are few studies regarding the ENSO impact on nitrogen cycling. One previous study compared the abundance of nitrogen cycling genes (*nir*KS, *nrf*A, *amo*A, and 16S anammox) from a La Niña period in 2018 to an El Niño period in 2019 in the ETNP (39). This study reported clear ENSO-phase–dependent differences for several genes, such as higher archaeal *amo*A and *nir*K abundances during El Niño and higher anammox 16S rRNA during La Niña. However, these gene-specific responses varied across stations from coastal to offshore regions, indicating that ENSO forcing influences nitrogen cycling gene distributions in a spatially heterogeneous manner rather than through uniform basin-scale responses (39). Another isotope-based study found that the source and fluxes of N_2_O were different between La Niña and El Niño in the ETSP (38). During La Niña in 2011, higher N_2_O fluxes were observed, and N_2_O was mainly generated by incomplete denitrification together with ammonia oxidation, while during El Niño (2010), N_2_O was likely primarily produced by AOA (38). Other studies also observed that a significant reduction of N_2_O emissions and a lower maximum of denitrification rates were associated with El Niño conditions in the ETSP, mainly because of the deepening of the upper boundary of the OMZ (35, 36).

**Fig 9:**
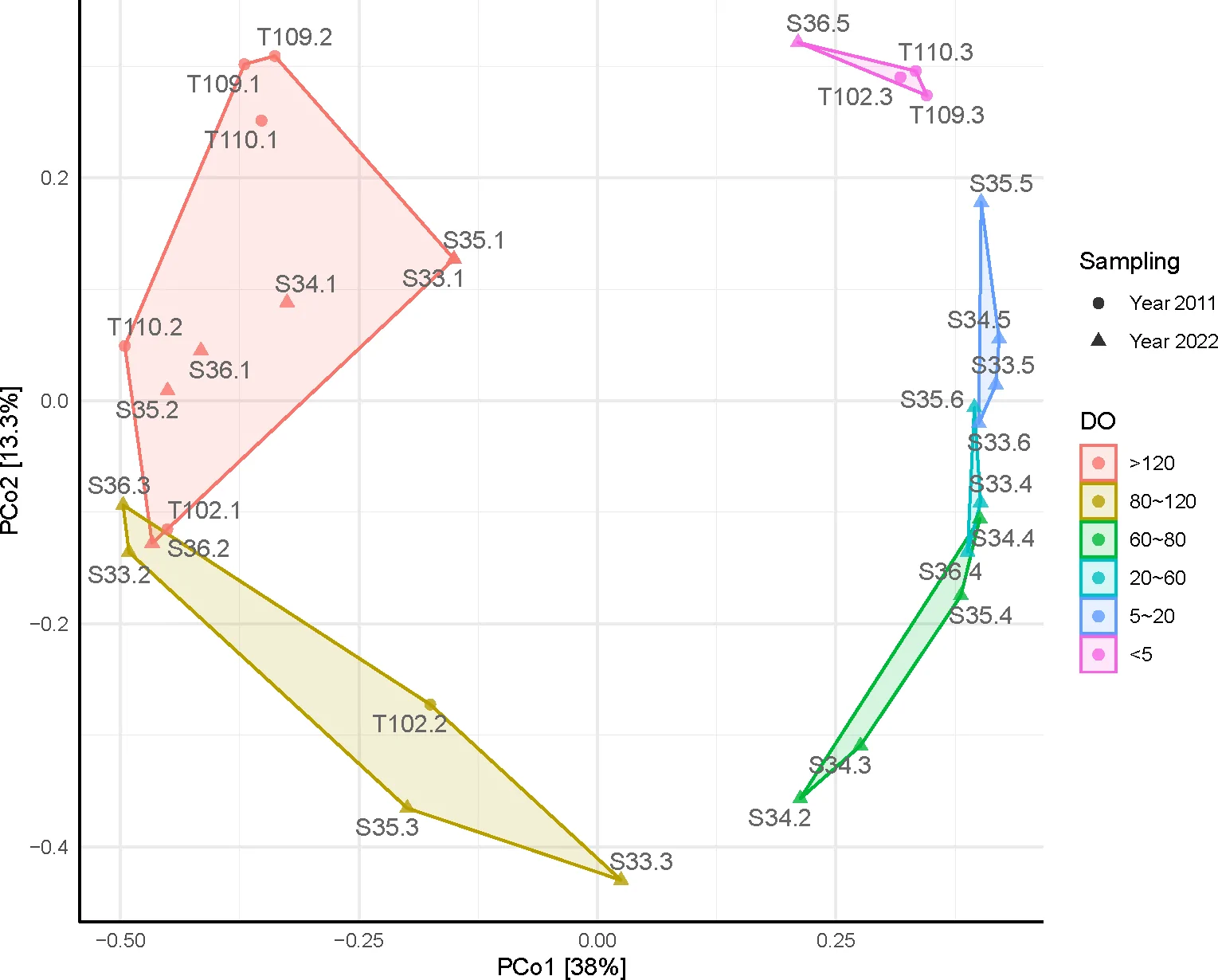
Principal coordinates analysis (PCoA) plot depicting microbial nitrogen metabolism composition from various ranges of DO concentrations (µmol kg^−1^) from both *Tara Oceans* and SO287 data sets in the ETP.

The comparison of our data set and *Tara Oceans* data set sheds new light on the temporal variability of microbial metabolisms in the ETP. It is noteworthy that the nitrogen functional groups did not show significant differences in the two La Niña events (Fig. 8), although the period between the two events was eleven years. The PCoA plot and RDA plot illustrate that nitrogen-cycling functional groups cluster more distinctly by spatial variations (e.g., DO gradients) than by sampling year (2022 vs 2011) (Fig. 9; Fig. S12). This pattern suggests that environmental heterogeneity across regions exerts a stronger influence on nitrogen metabolic taxa than temporal variability. The quality and availability of organic matter might be largely variable in the ETP, causing temporal differences in the relative abundance of certain genes of nitrogen cycle processes, especially those related to N_2_ loss and N_2_O production. However, because ENSO can modify the magnitude and spatial extent of the oxygen gradient, and our comparison includes only two La Niña events, further time-series sampling would be required to determine whether these patterns are maintained across different ENSO phases.

### Summary

We explored how deoxygenation might impact the microbial diversity, community composition, and nitrogen cycling genes in the ETP by comparing two La Niña years. We discovered a decrease in microbial diversity and total abundance, coupled with an elevated gene abundance of many nitrogen cycle pathways, along with a decline of DO in the OMZ. This indicates that deoxygenation would cause a loss of microbial abundance and diversity but an increase in the abundance of nitrogen cycle pathways. Furthermore, the relative abundance of many abundant microbial groups shifted at a DO threshold of 80 μmol kg^−1^. Nitrogen turnover processes exhibit strong DO-driven layering: complete nitrification dominates at DO at 5–120 µmol kg^−1^, while nitrate reduction prevails at DO < 20 µmol kg^−1^, transitioning to canonical denitrification, DNRA, and anammox at DO ~5 µmol kg^−1^. This also implies the primary source of N_2_O from either canonical denitrification or nitrification under different DO gradients. Moreover, deoxygenation reinforces many key microbial functional groups, such as AOA driving nitrification and potential N_2_O production, Nitrospina dominating nitrite oxidation, Gammaproteobacteria driving canonical denitrification, and Marinimicrobia and SAR11 dominating nitrate reduction. By comparing samples from the *Tara Oceans* campaign in 2011 and the SO287 campaign in 2022, which took place during comparable La Niña events, we found microbial alpha diversity and composition in the ETP did not shift significantly. However, we observed a significant decrease of SAR11 and an increase of Poseidoniia in both the euphotic layer and the OMZ core of the ETP in 2022 compared with 2011. We did not observe substantial shifts in nitrogen cycle relevant functional groups. However, in suboxic waters, more relative abundances of N_2_ loss associated genes were detected in 2022 than in 2011. Our study presents a comprehensive view of the impact of deoxygenation on microbial setting throughout the ETP OMZ water column during the La Niña events in 2011 and 2022, thus shedding new light on the temporal variability of microbial groups and functions in the ETP under both deoxygenation and ENSO dynamics. However, additional studies under different ENSO phases are needed to complete our picture. Moreover, we suggest that biogeochemical modeling studies should include the effects of climatic forcing on various biogeochemical cycles, such as the nitrogen cycle.

## Data Availability

Metagenomic sequence data generated in our study have been archived in the NCBI Sequence Read Archive (SRA) database under the Bioproject PRJNA983277 from SRR24907232 to SRR24907253.

## SUPPLEMENTAL MATERIAL

Supplemental figures msystems.00893-26-s0001.docx Fig. S1 to S10.

Supplemental tables msystems.00893-26-s0002.xlsx Tables S1 to S5.

ASM does not own the copyrights to Supplemental Material that may be linked to, or accessed through, an article. The authors have granted ASM a non-exclusive, world-wide license to publish the Supplemental Material files. Please contact the corresponding author directly for reuse.
