# Supplementary material for: Microbial diversity and nitrogen cycling across oxygen gradients in the eastern tropical Pacific during two La Niña years
Source: mSystems. 2026 Sep 21;11(10):e00893-26. doi: 10.1128/msystems.00893-26 (PMC13712833; doi:10.1128/msystems.00893-26)
Supplement: Supplementary file 1 [file msystems.00893-26-s0001.docx]

**Supplementary Information**

 Figure S1. Spatial profiles of physicochemical variables crossing the sampling regions and T-S diagram for the four stations of this study in the ETP.

Ocean Niño index

| 2-month "seasons" along the year | 2011 | 2015 | 2016 | 2019 | 2022 |
| --- | --- | --- | --- | --- | --- |
| DJ | -1.86 | 0.2 | 1.9 | 0.1 | -1.06 |
| JF | -1.64 | 0.1 | 1.8 | 0.5 | -0.96 |
| FM | -1.82 | 0.2 | 1.3 | 0.8 | -1.28 |
| MA | -1.86 | 0.3 | 1.3 | 0.3 | -1.76 |
| AM | -1.35 | 1 | 1.2 | 0.2 | -1.88 |
| MJ | -1.13 | 1.9 | 0.4 | 0.4 | -2.07 |
| JJ | -0.87 | 1.8 | -0.5 | 0.3 | -2.1 |
| JA | -0.89 | 2 | -0.3 | 0.3 | -1.79 |
| AS | -1.18 | 2.2 | -0.3 | 0.2 | -1.76 |
| SO | -1.43 | 2.2 | -0.5 | 0.3 | -1.71 |
| ON | -1.33 | 1.9 | -0.5 | 0.5 | -1.48 |
| ND | -1.24 | 1.9 | -0.4 | 0.4 | -1.25 |

Figure S2. The by-monthly Multivariate EI Niño/Southern Oscillation (ENSO) index (MEI.v2) of the years 2011 (strong La Niña), 2015-2016 (strong El Niño), 2019 (neutral), 2022 (strong La Niña), over the tropical Pacific basin (30°S-30°N and 100°E-70°W). El Niño/Southern Oscillation (ENSO) state is divided by neutral (|index| < 0.5), weak (0.5 < |index| < 1.0), and strong (|index| > 1.0) states. Months of January, April and May are highlighted. Our sampling period (January 2022) and *Tara Oceans* sampling period (April/May 2011) are in weak La Niña periods. Data from <https://psl.noaa.gov/enso/mei/>.

Figure S3. Model-derived environmental profiles over different ENSO phases within the framed region (polygon (86.450968W, 5.548545S; 84.415955W, 5.604299S; 84.165063W, 2.452124N; 86.339461W, 2.480001N)). The polygon region covers the sampling stations of both SO287 and *Tara Oceans*. Data from EU Copernicus Marine Service information - https://marine.copernicus.eu. Sea water temperature and salinity are from product of Global Ocean Physics Reanalysis (model: GLOBAL_MULTIYEAR_PHY_001_030; DOI:  <https://doi.org/10.48670/moi-00021>), which data were taken from the period of 1993 to 2026 with a 0.083° × 0.083° spatial resolution. Oxygen and nitrate are from product of Global Ocean Biogeochemistry Hindcast (model: GLOBAL_MULTIYEAR_BGC_001_029, DOI: <https://doi.org/10.48670/moi-00019>), which data were taken from the period of 1993 to 2026 with a 0.25° × 0.25° spatial resolution

Figure S4.a) for sea surface temperature (SST) (◦C) and b) for Chlorophyll-a (Chla) concentration (mg/m^3^) on January and April/May of four years covering two different ENSO phases, including strong La niña years (2011 and 2022) and strong El niño years (2015 and 2016). Data obtained from MODIS-Aqua; <https://giovanni.gsfc.nasa.gov> (SST from Aqua MODIS Level-3 Global Mapped 11µm Daytime Sea Surface Temperature (SST) Data, version R2019.0, 8-daily 4km resolution; Chla from Aqua MODIS Level-3 Global Mapped Chlorophyll (CHL) Data, version 2022.0, 8-daily 4km resolution).

Figure S5. Relative abundance of microbial families based on various depth niches in the ETP collected during SO287 cruise (a) and *Tara Oceans* projects (b).

Figure S6. Linear correlations between DO concentration and the relative abundance of the top 15 microbial classes. The dashed lines indicate the confidence interval.

Figure S7. Linear correlations between DO concentration and the relative abundance of the top 14 microbial families. The dashed lines indicate the confidence interval.

Figure S8. Principle coordinate analysis (PCoA) plot depicting microbial community composition from various ranges of DO concentrations (µmol kg^-1^) across four stations (S33-S36) in the ETP.

a)

b)

Figure S9. The rarefying curve of classified reads from both metagenome datasets. a) 22 metagenome datasets from this study with the minimum sequencing reads of 7358571, b) 9 metagenome datasets from Tara Ocean projects with the minimum sequencing reads of 19375119.

Figure S10. The comparison of the alpha diversity (after rarefication of reads) of microbial community (Chao1, Shannon, Simpson) between SO287 and *Tara Ocean* datasets. (* indicates the significance, **: p<0.01, ***: p<0.001, ****: p<0.0001; ns indicates no significance)

Figure S11. The comparison of the alpha diversity (before rarefication of reads) of microbial community (Chao1, Shannon, Simpson) between SO287 and *Tara Ocean* datasets. (* indicates the significance, ***: p<0.001; ns indicates no significance)

Figure S12. Redundancy Analysis (RDA) plot depicting microbial nitrogen metabolism gene composition constrained by environmental variables from both *Tara Oceans* (Year 2011) and SO287 (Year 2022) expeditions in the ETP. The RDA model explained 68.9% of total community variation (adjusted R² = 0.59). The first two constrained axes accounted for 77% of the explained variance. The ordination was performed on Hellinger-transformed gene abundance data with environmental parameters. Environmental vectors indicate the direction and strength of correlations between variables and microbial nitrogen metabolism gene composition.

Figure S13. Vertical distribution of heterotrophic bacteria in the sampling stations in the ETP.
